# Ligands for Intestinal Intraepithelial T Lymphocytes in Health and Disease

**DOI:** 10.3390/pathogens14020109

**Published:** 2025-01-23

**Authors:** Akanksha Hada, Zhengguo Xiao

**Affiliations:** Department of Animal and Avian Sciences, University of Maryland, College Park, MD 20742, USA; hada@umd.edu

**Keywords:** mucosal immunity, intestinal immunity, intraepithelial lymphocytes, IELs, immune tolerance, gut epithelial barrier, ligand-receptor interactions, T cell receptor signaling, TCR, IL-15

## Abstract

The intestinal tract is constantly exposed to a diverse mixture of luminal antigens, such as those derived from commensals, dietary substances, and potential pathogens. It also serves as a primary route of entry for pathogens. At the forefront of this intestinal defense is a single layer of epithelial cells that forms a critical barrier between the gastrointestinal (GI) lumen and the underlying host tissue. The intestinal intraepithelial T lymphocytes (T-IELs), one of the most abundant lymphocyte populations in the body, play a crucial role in actively surveilling and maintaining the integrity of this barrier by tolerating non-harmful factors such as commensal microbiota and dietary components, promoting epithelial turnover and renewal while also defending against pathogens. This immune balance is maintained through interactions between ligands in the GI microenvironment and receptors on T-IELs. This review provides a detailed examination of the ligands present in the intestinal epithelia and the corresponding receptors expressed on T-IELs, including T cell receptors (TCRs) and non-TCRs, as well as how these ligand-receptor interactions influence T-IEL functions under both steady-state and pathological conditions. By understanding these engagements, we aim to shed light on the mechanisms that govern T-IEL activities within the GI microenvironment. This knowledge may help in developing strategies to target GI ligands and modulate T-IEL receptor expression, offering precise approaches for treating intestinal disorders.

## 1. Introduction

The epithelial tissues lining the body’s surfaces form a vital barrier. They serve as the first line of protection against external threats while enabling the movement of ions, nutrients, and water across the epithelium. This role is especially critical in the intestinal epithelium, as it is continually exposed to a complex mixture of dietary antigens, commensal microorganisms, and potential pathogens [1,2]. In this challenging environment, the immune system must maintain tolerance to benign stimuli while mounting effective responses against the pathogens [2,3]. Among the key players are the intestinal intraepithelial T lymphocytes (T-IELs), a specialized population of immune cells residing within the intestinal epithelial layer [4]. These cells are strategically positioned and respond rapidly to changes in the gut microenvironment, playing an important role in maintaining intestinal homeostasis and coordinating immune responses [4,5].

T-IELs can be broadly categorized into two main subsets: natural T-IELs and induced T-IELs, each with distinct developmental origins and functional characteristics. Natural T-IELs, which include TCRαβ+CD8αα+ and TCRγδ+ subsets, originate either directly from the thymus or extrathymic compartments [6]. Although the mechanisms underlying their development are not fully understood, these cells are considered pre-activated due to their recognition of self-antigen ligands and expression of activation markers during their ontogeny [7,8,9,10]. In contrast, induced T-IELs, comprising TCRαβ+CD8αβ+ and TCRαβ+CD4+ subsets, arise from conventional naive T cells that are activated by retinoic acid-producing dendritic cells in gut-associated lymphoid organs (GALT) such as mesenteric lymph nodes and Peyer’s patches [11,12,13,14,15,16,17]. The activation of precursors of both natural and induced T-IELs upregulates gut-homing molecules such as α4β7 integrin and CCR9, facilitating their migration to the lamina propria of the intestinal mucosa [18,19,20,21,22,23,24,25]. Once there, these T-IELs undergo phenotypic and functional adaptations to establish their tissue-resident status within the epithelial layer. A key step in this process is a TGFβ-mediated integrin switch, where the expression of α4β7 integrin is downregulated while αEβ7 is upregulated, facilitating their migration into the epithelium [11,18,26]. Concurrently, T-IELs undergo significant transcriptional reprogramming, which reduces the expression of genes involved in lymph node trafficking (e.g., CD62L and CCR7) and tissue egress (e.g., S1PR1, KLF2, and CXCR1) for their epithelial residency [27]. Additionally, T-IELs adopt a unique transcriptional profile driven by IL-15-regulated transcription factors, including T-bet and Runx3 [27]. These factors are crucial in programming the functional characteristics of T-IELs and guiding their terminal differentiation. As a result, T-IELs acquire the capacity to produce cytokines such as IFNγ and effector molecules such as granzymes, equipping them to fulfill their regulatory and protective roles in the GI microenvironment [27].

**Figure 1:**
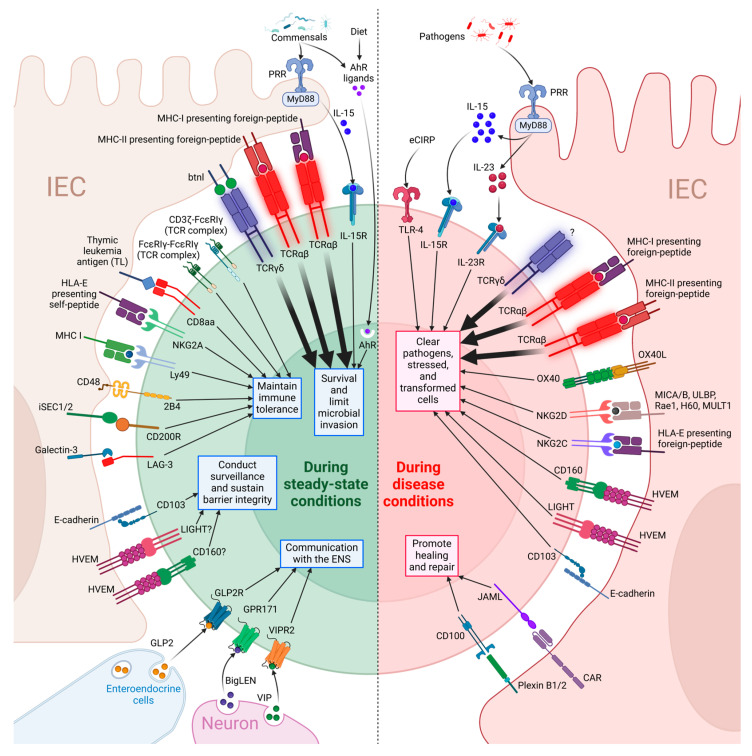
Availability of potential ligands and the possible expression of T-IEL receptors in steady-state and disease conditions. This diagram illustrates the crucial roles of ligand availability and receptor expression on T-IELs within the intestinal epithelial environment under both steady-state (**left**) and disease (**right**) conditions. Under steady-state conditions, specific ligands and their corresponding receptors are essential for preserving T-IEL populations, preventing microbial invasion, maintaining immune tolerance, conducting immune surveillance, sustaining the integrity of the epithelial barrier, and communicating with the enteric nervous system, ultimately maintaining homeostasis. In contrast, during disease conditions, changes in ligand availability and ligand-receptor interactions aid in eliminating pathogens, stressed and malignantly transformed cells, as well as in promoting tissue repair and healing. TCR, T cell receptor; PRR, pattern recognition receptor; TLR, toll-like receptor; AhR, aryl hydrocarbon receptor; IL, interleukin; MHC, major histocompatibility complex; HVEM, herpesvirus entry mediator receptor; GLP, glucagon-like peptide; VIP, vasoactive intestinal peptide; eCIRP, extracellular cold-inducible RNA-binding protein; JAML, junctional adhesion molecule-like; CAR, coxsackievirus and adenovirus receptor. Created in BioRender.com (accessed on 15 January 2025).

The functional diversity and specificity of T-IELs are fundamentally governed by real-time interactions between available ligands and expressed receptors on these cells within the epithelial layer, allowing them to rapidly adapt their responses to the dynamic intestinal milieu. As illustrated in Figure 1, the receptors on T-IELs can be broadly categorized into two main types: T cell receptors (TCRs) and non-TCR receptors. TCRs serve as the primary sensors for antigen recognition and initiating immune responses [28]. In peripheral T cells, TCRαβ recognizes pathogen-derived peptides presented by major histocompatibility complex (MHC) molecules [29,30], whereas TCRγδ is generally considered not to be restricted to MHC [31]. Within the intestinal epithelia, the specific ligands for the TCRs of natural T-IELs are not fully characterized. However, natural T-IELs with TCRαβ are known to interact with classical and non-classical MHC molecules [11,32,33,34,35,36,37], while TCRγδ in the TCRγδ+ subset (Vγ7+ in mice and Vγ4+ in humans, which constitute the major population) binds to butyrophilin-like (btnl) molecules expressed by IECs in an MHC-independent manner [10,11,26,38]. Although induced T-IELs are derived from conventional T cells, their TCRs are known to recognize not only pathogen-derived but also commensal-derived antigens presented by the MHCs, reflecting their unique functional adaptation to the gut [11,26]. Non-TCR receptors, on the other hand, fine-tune TCR signaling and can also potentially regulate T-IEL functions independently. These include cytokine receptors, co-stimulatory and co-inhibitory receptors, as well as pattern recognition receptors (PRRs), all depicted in Figure 1 along with their potential ligands in the GI microenvironment [4,26,39,40,41,42,43,44,45]. It is important to note that these receptors, reported in the literature, are dynamically regulated by signals within the GI microenvironment. Consequently, not all T-IELs express all these receptors simultaneously or at all times. The interaction of these receptors with specific ligands during various physiological and pathological states initiates cascades of intracellular signaling events [26,27,35,40]. The cumulative effect of these interactions collectively defines the specific functional profile of T-IELs, enabling them to respond appropriately to the ever-changing intestinal environment. This allows T-IELs to maintain intestinal homeostasis under steady-state conditions and mount robust immune defenses during infections, while potentially contributing to pathogenesis in chronic illnesses.

Although ligand-receptor interactions are crucial for determining T-IEL function, there has been a lack of detailed analysis of the various ligands in the GI microenvironment and their specific interactions with T-IEL receptors. This review seeks to offer an overview of known ligand-receptor interactions and their impacts on T-IEL functions in both steady-state and pathological conditions, as illustrated in Figure 1. We explore T-IELs under steady-state conditions, focusing on how the availability of GI ligands and T-IEL receptor expression enable them to sense microbial components, modulate receptor expression and associated signaling pathways to promote immune tolerance, sustain survival, support immune surveillance, maintain epithelial barrier integrity, and potentially facilitate crosstalk with the enteric nervous system (Figure 1; left). In disease contexts, we examine GI ligands and T-IEL receptor expression, highlighting ligand-receptor interactions and their roles in pathogen clearance, the pathogenesis of chronic conditions such as celiac disease, inflammatory bowel disease, and cancer, as well as tissue repair (Figure 1; right).

## 2. T-IEL Ligands in Steady-State Condition

T-IELs are actively engaged in responding to ligands within the GI epithelia under steady-state conditions, which is crucial for maintaining intestinal homeostasis (Figure 1; left). These cells can selectively express a diverse array of receptors that interact with specific ligands available in the intestinal microenvironment during steady-state conditions. These interactions involve TCR signaling [27,46,47,48], microbial ligands signaling through Pattern Recognition Receptors (PRRs) on IECs [39,49], cytokine signaling via local mediators such as IL-15 [26,39,41,42,43], dietary influences from AhR ligands [26,35,43,50,51], and pathways mediated by other co-receptors [4,35]. T-IEL functions include preventing potential pathogen invasion by sensing microbial ligands, regulating receptor expression to promote immune tolerance, and supporting T-IEL survival. Additionally, they contribute to immune surveillance, maintain tight junctions, and facilitate communication with the enteric nervous system. Overall, the ligand-receptor interactions collectively create a complex network of signals that sustain intestinal homeostasis within the GI epithelia, as shown in Figure 1 (left).

### 2.1. Monitoring Lumen Microbes

Microbial components are sensed by T-IELs directly through their TCRs or indirectly via IECs [27,39,46,47,48,49]. These interactions drive cytokine production such as IL-15 by IECs [26,39,41,42,43], effector molecule expression, and antimicrobial peptide (AMP) release [52,53,54,55], ultimately strengthening the immune surveillance and regulatory functions of T-IELs to protect the epithelial barrier.

#### 2.1.1. Direct Sensing via TCRs

The sensing of microbial components through TCRs is a critical aspect of immune surveillance in the intestinal epithelium. Induced T-IELs, including both TCRαβ+CD4+ and TCRαβ+CD8αβ+ subsets, appear to play a key role in this process by recognizing and responding to GI lumen microbial antigens that are constantly sampled by antigen-presenting cells (APCs) during steady-state conditions [27,46,47,48,56,57,58]. Despite the diverse luminal antigens at epithelial barriers, T-IELs exhibit limited TCR diversity, potentially allowing them to recognize conserved microbial or dietary components [27]. While some mechanisms and antigens are identified, our overall understanding of the process that drives this selective process is still limited. For instance, TCRαβ+CD4+ T-IELs can recognize specific microbial enzymes such as β-N-acetylhexosaminidase from Bacteroidetes through their TCRs, which leads to proliferation and protection against inflammation in colitis models [27,46]. Furthermore, the interaction between TCRs on CD4+ T-IELs and MHC-II molecules presenting microbial peptides on IECs is crucial for their activation and expansion. This interaction also supports subsequent IL-10 production, which is vital for effective microbial sensing and maintaining immune tolerance [47]. Additionally, MHC-II-dependent recognition of segmented filamentous bacteria (SFB) promotes the accumulation of SFB-specific TCRαβ+CD4+ T-IELs. These T-IELs are also capable of producing Granzyme B, which aids in the turnover of IECs [48]. Similarly, TCRαβ+CD8αβ+ T-IELs depend on TCR ligands for their survival and maintenance within the intestinal epithelium [56,57,58]. Although their specific ligands under normal physiological conditions remain undefined, variations in TCR Vβ usage, particularly in Vβ6, Vβ7, and Vβ11, have been observed in these T-IELs from mice under specific pathogen-free (SPF), germ-free (GF), and antigen-free diet conditions [58]. These findings suggest that TCR signaling in steady-state conditions may be influenced by interactions with intestinal microbiota in TCRαβ+CD8αβ+ T-IELs. Through these mechanisms, the TCRs on TCRαβ+CD4+ and TCRαβ+CD8αβ+ T-IELs seem to serve as crucial receptors for sensing microbial components in the intestinal environment, enabling a targeted immune response that maintains intestinal homeostasis under steady-state conditions.

#### 2.1.2. Indirect Sensing Through IEC-Mediated Cytokine Signaling

The sensing of potential pathogens by T-IELs also occurs indirectly through interactions with IECs, providing effective protection for the epithelial barrier. IECs express pattern recognition receptors (PRRs) such as TLR-2 and TLR-4, which detect pathogen-associated molecular patterns (PAMPs) from microbes and trigger the production of cytokines, particularly IL-15. This cytokine plays a crucial role in activating, proliferating, and modulating the functions of T-IELs [11,26,39,41,42,43,55]. For instance, TLR-2 deficient mice exhibit reduced numbers of various T-IEL subsets, including TCRαβ+CD8αα+, TCRαβ+CD8αβ+, and TCRγδ+ T-IELs, with the remaining T-IELs showing decreased activation and proliferation, along with increased apoptosis [39]. In the colon, bacteria from the Bacteroidales order stimulate IL-6 production by T-IELs through NOD2 and MyD88 signaling in IECs, contributing to protection against *Citrobacter rodentium* infection [49]. PRR signaling-dependent IL-15 production by IECs also stimulates TCRγδ+ and TCRαβ+CD8αβ+ T-IELs to produce antimicrobial peptides (AMPs) and enhances the cytotoxic activity of TCRαβ+ T-IELs [52,53,54,55]. This AMP production is crucial for preventing pathogen establishment and maintaining spatial separation between the microbiota and the intestinal epithelial surface under steady-state conditions [59]. While there is no evidence of T-IELs directly sensing microbes through their PRRs, TCRαβ+CD4+CD8αα+ T-IELs can eliminate infected IECs in response to damage-associated molecular patterns (DAMPs) such as extracellular cold-inducible RNA-binding protein (eCIRP) via TLR-4 [45]. However, it remains unclear whether these T-IELs can directly recognize microbial TLR-4 ligands such as lipopolysaccharide (LPS) [45]. This indirect sensing mechanism through cytokine receptor signaling complements the direct TCR-mediated recognition, providing a comprehensive surveillance system for maintaining intestinal homeostasis and protecting against potential pathogens.

Overall, the interaction of TCRs on T-IELs and PRRs on IECs with microbial-derived ligands provides essential signals that program T-IELs to prevent pathogen invasion. This programming modulates cytokine production, cytotoxic molecule release, and AMP secretion under steady-state conditions. While there is evidence of increased expression of intracellular peptidoglycan recognition receptors in TCRαβ+CD8+ T-IELs during bacterial infection [43] and TLR-4 on TCRαβ+CD4+CD8αα+ T-IELs [45], comprehensive data on PRR expression and functionality across different T-IEL subsets remains limited, contrasting with the well-documented situation in peripheral T cells [60]. Interestingly, TCRαβ+CD8αα+, TCRαβ+CD8αβ+, and TCRγδ+ T-IELs appear to rely more heavily on signals from IECs, particularly IL-15, while TCRαβ+CD4+ T-IELs depend more on their TCR signaling during steady-state conditions [39,49,52,53,54,55]. To enhance our understanding of pathogen invasion prevention by T-IEL subsets, further research is needed in several areas. These include exploring TCR-mediated signaling pathways beyond TCRαβ+CD4+ T-IELs, examining TLR expressions and functions within various T-IEL subsets, and investigating how different TLR stimulations in IECs indirectly activate distinct T-IEL populations.

### 2.2. Modulating Receptor Expression and Signaling Pathways for Immune Tolerance

T-IELs achieve immune tolerance in the GI epithelia through various means during steady-state conditions. Firstly, natural T-IELs in the intestine are equipped with tamed TCR signaling from birth, which is designed to balance responding to threats such as potential pathogens and tolerating normal gut contents such as commensals and dietary components. Modifications in the TCR complex and TCR signaling pathway reduce natural T-IELs’ reactivity [38]. Secondly, the regulated expression of both inhibitory and stimulatory co-receptors on both natural and induced T-IELs as well as the availability of their respective ligands in the intestinal microenvironment establishes a finely tuned system for ensuring controlled immune responses that are crucial for immune tolerance by enabling T-IELs to maintain vigilance without overreacting under steady-state conditions [4,35].

#### 2.2.1. Reduced TCR Signaling in Natural T-IELs

Natural T-IELs, including TCRαβ+CD8αα+ and TCRγδ+, are enriched in the intestine at birth, suggesting a pre-established role in neonatal immunity [61,62,63,64,65,66,67,68]. Although the intestinal microenvironment contains natural T-IELs’ TCR ligands, such as classical and non-classical MHC-I molecules as well as MHC-II molecules presenting self-antigens ligands for TCRαβ+CD8αα+ T-IELs, along with btnl ligands on IECs for TCRγδ+ T-IELs, TCR signaling remains suppressed under steady-state conditions [10,11,32,33,34,35,36,37,38]. This reduced TCR signaling is primarily achieved through a distinct CD3 complex configuration, composed of either CD3ζ-FcεRIγ heterodimers or FcεRIγ-FcεRIγ homodimers, which differs from the CD3ζ-CD3ζ homodimers expressed by conventional T cells [69]. CD3ζ typically has three immunoreceptor tyrosine-based activation motifs (ITAMs), whereas FcεRIγ has only one. ITAMs are found in the cytoplasmic tails of the T cell receptor complex and are crucial for initiating signal transduction [70]. Therefore, the CD3 complex having FcεRIγ has decreased potency and sensitivity of TCR signaling [71,72,73]. Moreover, natural T-IELs have significantly lower levels and reduced phosphorylation of Linker for Activation of T cells (LAT) protein as well as higher levels of LAT2, which further helps in decreasing the TCR signaling, compared to conventional TCRαβ+CD8αβ+ T cells and induced T-IELs [38]. LAT is an adaptor protein essential for TCR-mediated signaling and T cell development, acting as an anchoring point for the assembly of TCR signaling complexes upon activation [74]. Restoring LAT expression in natural T-IELs rescues TCR signaling, as indicated by increased phosphorylation of downstream TCR signaling molecules [38]. Furthermore, LAT2 plays a dominant negative role in TCR signaling by competing with LAT for binding partners [38,75,76], and knocking out LAT2 leads to increased IFNγ and TNFα production by natural T-IELs in vivo [38]. The reduced TCR signaling in natural T-IELs is functionally relevant, as it aligns with their tolerogenic role in the intestinal microenvironment. By suppressing TCR signaling, natural T-IELs might avoid unnecessary activation and pro-inflammatory responses to self-antigens and commensal microbiota, thus maintaining immune homeostasis and preventing tissue damage. For example, even though btnl molecules interact with the TCRs of TCRγδ+ T-IELs, this interaction is known to diminish their TCR expression and decrease the production of pro-inflammatory cytokines such as IL-6 and IFNγ [77,78]. Overall, the evidence suggests that even though natural T-IELs’ TCR ligands are present in the intestinal microenvironment, the modified TCR signalosome reduces their TCR signaling compared to peripheral T cells under steady-state conditions [38,79]. This mechanism should help natural T-IELs sustain self-tolerance within the gut and minimize the risk of inappropriate immune activation, harmful inflammation, or tissue damage in response to self-antigens and commensals.

#### 2.2.2. Regulation of Co-Receptor Expression and Signaling Pathways

T-IELs regulate co-receptor expression and their signaling adapters to maintain immune tolerance. In mice, this regulation occurs through a coordinated process involving the upregulation of co-inhibitory receptors such as CD8αα, CD200R1, 2B4, LAG-3, Ly49 family members, PD-1, CD39, CD73, CD160, CD96, and CD161. Simultaneously, there is a reduction in expression and weakened signaling of activating co-receptors, including CD2, CD5, CD28, and NKG2D [4,35]. Furthermore, the availability of ligands for these receptors in the GI microenvironment is finely regulated. This intricate balance of receptor expression, signaling strength, and ligand availability collectively contributes to a state of immune tolerance under normal physiological conditions.

A major inhibitory co-receptor CD8αα is expressed in the majority of T-IELs in mice; however, it is negligibly expressed in human T-IELs [40,44,80,81,82], indicating unique regulatory mechanisms in these species. Tissue ligands such as TGFβ, IL-15, retinoic acid, IL-27, and IFNγ help induce the CD8αα homodimer in T-IELs in mice [26,80,81,83]. The CD8αα binds Thymus-leukemia antigen (TL) that are highly expressed on IECs, and this CD8αα-TL interaction represses TCR signaling by redirecting CD8αα and its associated lck tyrosine kinase away from the TCR [26], which prevents the full activation of T-IELs [84]. Additionally, TL also induces the death of activated CD8αβ+ T-IELs that do not co-express CD8αα [84], thereby eliminating the chances of unwanted activation. The immunoregulatory effect of CD8αα-TL interaction is further supported by the fact that TL-deficient mice develop accelerated colitis in an inflammatory bowel disease (IBD) model [85], probably due to elevated TCR signaling in T-IELs. These effects likely control the balance between the anti-inflammatory and cytotoxic functions of T-IELs, especially during steady-state conditions, in mice. However, loss of TL expression only increases the proliferation of colonic CD8αα+ T-IELs, but not on small intestinal T-IELs [43], which mandates further research. Furthermore, CD8αα expression is rarely detected in human T-IELs [86], indicating that human T-IELs are regulated through different mechanisms that are discussed below.

Under steady-state condition, both of the natural T-IELs and TCRαβ+CD8αβ+ induced T-IELs highly express inhibitory receptors, including CD200R1, 2B4/CD244, LAG-3, PD-1, CD39, CD73, CD160, CD96 and Natural Killer (NK) receptors, such as CD94/NKG2A, CD161, members of the Ly49 family. Natural T-IELs express a wider variety of these inhibitory receptors than induced T-IELs [35,75,87]. iSEC1 and iSEC2, ligands of CD200R, and CD48, a ligand of 2B4, are expressed exclusively by secretory cell lineages such as tuft cells, goblet cells, Paneth cells, enteroendocrine cells, etc., in the GI epithelium [75,88,89]. The binding of these ligands to their respective co-receptors on T-IELs could suppress inflammatory cytokine production and cytolytic activity by activating T-IELs. There is higher phosphorylation of intracellular adapters of CD200R1 and 2B4 in natural T-IELs compared to conventional CD8+ T cells, indicating their inhibitory role during steady-state conditions [38]. However, the suppressed feature is also observed in T-IEL cultures, and T-IELs do not express any CD200R1 and 2B4 ligands [38]. This raises uncertainty about whether CD200R1 and 2B4 ligands play an active role in T-IEL immune tolerance under steady-state conditions. Moreover, galectin-3, a ligand of LAG-3, is predominantly expressed in IECs of the villus tips [90]. Galectin-3 knockout mice suffer from a more severe disease progression in a DSS–induced colitis model [91], indicating their regulatory role in the GI epithelia. Apart from galectin-3, IECs express MHC class II, which is also a canonical ligand of LAG-3 [92,93]. However, there was no difference in the response of T-IELs when cultured with or without anti-LAG-3 antibodies for 96 hours before stimulating it with anti-CD3 [38]. Additionally, ablation of TCR in TCRαβ+CD8αα+ T-IELs decreased the expression of LAG-3 [50]. This suggests that TCR stimulation by their respective ligands might be required to increase the expression of LAG-3, which then responds to LAG-3 ligands to regulate the immune responses of these T-IELs. Furthermore, T-IELs in mice expressing the inhibitory Ly49 receptors such as Ly49A and Ly49G2, which bind to MHC-I molecules expressed on IECs, show diminished responses when their TCR is activated. This reduced responsiveness is indicated by an absence of CD69 upregulation and lower production of components that are vital for the activation and functionality of these immune cells, such as chemokines like MIP-1α and lymphotactin [94]. On the other hand, T-IELs that do not express these Ly49 receptors, exhibit a more robust response to TCR stimulation [94]. These findings suggest that ligands for Ly49 inhibitory NK receptors are one of the candidates for being a regulating factor for T-IELs in steady-state conditions.

Apart from the expression of co-inhibitory receptors and the availability of their respective ligands, T-IELs typically lack or display reduced expression of co-stimulatory receptors. Additionally, their signaling is impaired and the co-stimulatory ligands are minimally expressed under steady-state conditions. For instance, natural T-IELs exhibit downregulated expression of TCR co-stimulatory molecules including CD2, CD5, and CD28 [27,69]. Moreover, TCRαβ+CD8αβ+ T-IELs in mice do not express NKG2D, a type II transmembrane C-type lectin activating NK receptor, under steady-state conditions [40]. Distinct in humans, while TCRαβ+CD8αβ+ T-IELs express NKG2D, they lack ITAM adapter molecules such as DAP12 that couple with NK receptors and mediate intracellular activation signals, thereby reducing their functional capabilities, including proliferation and cytokine secretion [95,96,97,98]. Thus, the TCR might be the only activating receptor capable of inducing cell proliferation and cytokine production in healthy human TCRαβ+CD8αβ+ T-IELs [40]. Experimental evidence has shown that DAP12 expression is reduced under steady-state conditions but upregulated under pathological conditions [96,97]. Furthermore, under steady-state conditions, NKG2D receptor ligands such as MHC class I polypeptide-related sequence A/B (MICA/MICB) and UL16-Binding Proteins (ULBP) family members are either present at low levels or absent on IEC [99,100]. These lines of evidence suggest a distinct mechanism of tolerance in humans and mice.

In summary, the regulation of T-IELs through ligands that promote immune tolerance is crucial for maintaining gut homeostasis. Key to this regulation is the subdued TCR signaling in natural T-IELs. Moreover, the regulated expression of various co-receptors on T-IELs, influenced by the availability of specific ligands within the intestinal microenvironment, forms a sophisticated regulatory network. Additionally, significant species-specific differences in ligand interactions, particularly in the expression and function of co-receptors such as CD8αα and NKG2D in mice and humans, underscore unique regulatory mechanisms across species. Future research could focus on the roles and ligands of less understood co-receptors on T-IELs, including PD-1, CD39, CD73, CD160, CD96, and CD161 [35,75,87]. Additionally, investigating how altered TCR complex configurations affect TCR signaling in natural T-IELs during events such as pathogen invasion is important. Furthermore, it is important to explore the factors that regulate the expression of various inhibitory and stimulatory co-receptors on both natural and induced T-IELs, and the availability of their ligands under different conditions to understand their in-depth role in the intestine.

### 2.3. Essential Ligands for T-IEL Survival

The survival of T-IELs within the GI epithelia is facilitated by a complex network of ligand-receptor interactions, involving various receptors, such as TCRs [4,32,33,34,35,37], cytokine receptors [11,26,39,41,42,43,44,55,98,101], nuclear receptors [26,35,43,50,51], and other signaling co-receptors [102,103,104,105,106].

#### 2.3.1. TCR and Co-Receptor Signaling Requirements

Receptor-mediated signaling such as through TCR and β1 integrins has been known to be important in T-IEL maintenance. TCR ligands are dispensable for TCRαβ+CD8αα+ and TCRαβ+CD4+ T-IELs’ survival but not for TCRγδ+ and TCRαβ+CD8αβ+ T-IELs, in steady-state conditions. For instance, although TCRαβ+CD8αα+ natural T-IELs have heterogenous TCRs that recognize either classical or nonclassical MHC molecules presenting self-antigen ligands [4,32,33,34,35,37], they can survive without surface expression of their TCR in the steady-state [20]. Moreover, the ablation of the TCR or long-term antigen withdrawal does not affect the maintenance of TCRαβ+CD4+ T-IELs [79,107]. Therefore, these subsets do not require TCR signaling for their survival once they reach the GI epithelium [20,79,107]. In contrast, TCRαβ+CD8αβ+ T-IELs do require TCR stimulation for their maintenance [56,57,58]. Their frequency decreases over time as encounters with antigen ligands on MHC molecules diminish, indicating their reliance on their TCRs for maintenance [56,57]. In the case of TCRγδ+ T-IELs, they are not restricted to any MHC molecules and their maintenance is independent of microbial or dietary antigens [11,43]. Instead, the predominant TCRγ7+ subset in mice relies on their TCRs binding to btnl1/6 on the IECs for their maintenance [108,109]. Similarly, in humans, the TCRγδ+ T-IEL population is primarily Vy4+ and is dependent on the expression of BTNL3/8 on IEC [110]. Btnl expression on IECs is crucial for maintaining the TCRγ7+ T-IEL subset, as its absence completely eliminates this population [108,109,110,111]. IECs express the HVEM (Herpesvirus Entry Mediator) receptor, a member of the TNF superfamily, whose ligand, LIGHT, enhances the epithelial production of basement membrane proteins, such as collagen IV. This basement membrane protein is known to interact with β1 integrins on T-IELs, promoting their survival in vitro [102]. This is further supported by the fact that the absence of β1 integrin leads to a reduction in TCRαβ+ T-IELs in vivo [102].

Overall, the TCRs and co-receptors are essential for sustaining T-IEL populations within the gut in a steady state. However, TCRαβ+CD8αα+ and TCRαβ+CD4+ T-IELs do not require TCR stimulation for their survival, making them notable exceptions.

#### 2.3.2. Microbial Signals and Cytokine-Dependent Survival Signals

Microbial components and local cytokines in the intestinal microenvironment are crucial for the maintenance of T-IELs. Microbial colonization seems to selectively induce clonal expansion of TCRαβ+ T-IEL subsets including TCRαβ+CD8αα+, TCRαβ+CD8αβ+, and TCRαβ+CD4+, as oligoclonal TCRs are found on these T-IELs in the presence of microbes [40,112,113,114,115,116]. Additionally, the total number of T-IELs is significantly less in Germ-Free (GF) and antibiotic-treated mice as compared to that in specific pathogen-free (SPF) mice [55,117], and this number increases after microbial colonization in GF mice [118], which almost reaches the level in conventional mice [67]. This expansion of T-IELs relies on the expression of PRRs, including TLR and NOD-like Receptors on IECs [11,26,39,41,42]. These PRRs detect intestinal microbes, such as *Clostridia*, *Lactobacillus acidophilus*, *Bifidobacterium longum*, segmented filamentous bacteria (SFB), and viruses, which in turn result in the production of IL-15 by IECs, aiding in the maintenance of all T-IELs [11,26,39,41,42,43,44,55,101]. The critical role of IL-15 in T-IEL maintenance is evidenced by the marked reduction of T-IELs in IL-15 knockout mice [39,119]. The depletion of TLR-2 or NOD2 decreases the number of these T-IEL subsets due to impaired IL-15 expression [39,119].

#### 2.3.3. Dietary and Microbial-Derived Components

Dietary and microbial-derived components also play an essential role in the maintenance of T-IELs in GI epithelia. The Aryl Hydrocarbon Receptor (AhR) is a nuclear receptor that is highly expressed in T-IELs, and tryptophan-derived ligands, either dietary (indole-3-carbinol from cruciferous vegetables) or microbial (tryptophan metabolites from commensals such as *Lactobacillus reuteri*), are crucial for the survival and maintenance of all T-IEL subsets [26,35,43,50,51]. This is further supported by the fact that AhR-deficient mice or those lacking dietary AhR ligands have fewer T-IELs [120,121,122]. Moreover, AhR-deficient mice develop more severe colitis following DSS treatment than wild-type mice, and AhR ligands have been shown to relieve this colitis [120,123], possibly due to sustained T-IEL numbers. Additionally, dietary glutamine is known to aid in IL-22-AhR-dependent and IL-15-independent maintenance of the T-IEL population [124]. Furthermore, intestinal commensals convert dietary unsaturated fatty acids such as linoleic acid into conjugated linoleic acid isomers. These isomers help maintain TCRαβ+CD4+CD8αα+ T-IEL populations through unknown mechanisms, as evidenced by the decrease in this T-IEL subset when fatty acid isomerization pathways are genetically abolished [125].

Overall, T-IELs depend on a variety of receptors for their survival in the gut, including TCRs, membrane proteins, cytokines receptors, and nuclear receptors. Given the complexity of the intestinal epithelia, it is likely that additional receptors also contribute to the survival and maintenance of these T-IELs. Future research could focus on understanding if TCRαβ+CD8αα+ T-IELs recognize any specific self-peptide ligand in the gut and the effects of these interactions [38]. Conditional deletion of MHC molecules, specifically in the IEC in vivo, could shed more light on the functional outcomes of these interactions on TCRαβ+ T-IELs. Moreover, btnl on IECs is essential for the survival of TCRγδ+ T-IELs. However, it is still unclear how btnl expression is regulated on IECs, which signaling pathways it activates, whether other TCR ligands for TCRγδ+ T-IELs exist in the GI epithelia, and how these interactions affect TCRγδ+ T-IEL maintenance under steady-state conditions. Moreover, how T-IELs with specific TCR repertoires are selected for clonal expansion in different subsets of TCRαβ+ T-IELs, especially since this expansion seems to be TCR-independent is still not well characterized. Additionally, the maintenance mechanisms of TCRγδ+ T-IELs in the absence of microbes and microbe-dependent IEC-derived IL-15 remain poorly understood.

### 2.4. T-IEL Surveillance and Barrier Maintenance

Microbial signals mediate T-IEL’s motility and patrolling behavior. This surveillance is supported by occludin, a tight junction component, along with E-cadherin and EpCAM, which are cell adhesion molecules on T-IELs that enable crucial interactions with IECs [5,75,106,126,127]. Glucose availability also ensures swift T-IEL migration, regulates barrier permeability, and maintains epithelial integrity [5]. HVEM ligands and GLP2R (glucagon-like peptide 2 receptors) on T-IELs further might enhance surveillance and repair functions, hinting at a sophisticated network that sustains immune homeostasis and intestinal integrity [75,102,103,104].

Local IL-15 produced by IECs in response to microbial PRR signals mediate TCRγδ+ T-IELs surveillance behavior. This is indicated by experiments where the inhibition of IL-2Rβ, a subunit of IL-15 receptor, in these T-IELs significantly attenuated the basal motility of these cells, resulting in their idling within the lateral intercellular space during early invasion of pathogens such as *Salmonella typhimurium* [5,26,128]. Moreover, occludin expressed on TCRγδ+ T-IELs mediates their rapid migration within the space between the epithelial layer and the basement membrane by contacting IECs, which helps them cover the entire intestinal epithelium within a few hours [5,106,126]. Occludin’s tight associations with the presence of ZO-2 in T-IELs could enhance the intestinal epithelial barrier function by contributing to the formation and maintenance of tight junctions and could regulate the permeability of the epithelial barrier, ensuring proper movement of T-IELs between neighboring cells while also maintaining the integrity of the intestinal lining [75,129]. Additionally, this migration is dependent on glucose availability, as systemic administration of glucose-inhibitor, 2-deoxy-glucose, inhibited this movement in TCRγδ+ T-IELs [5]. Moreover, epithelial retention of TCRγδ+ T-IELs within the lateral intercellular spaces is partly mediated by the direct interaction of T-IEL’s CD103 with E-cadherin on IEC [106]. E-cadherin and EpCAM, expressed on T-IELs, facilitate interactions with IECs and help in maintaining the structural integrity of epithelial tight junctions and regulating paracellular permeability across epithelial cells [75,127]. Furthermore, since T-IELs reside within the epithelial layer and do not recirculate, endothelial cell adhesion molecules such as PECAM-1 and CD62L, that facilitate naive T cell migration into secondary lymphoid organs, are not expressed on T-IELs [75]. 

Besides junction proteins, other receptors also seem crucial for T-IEL’s surveillance and tight junction functions. HVEM expressed on IECs has been shown to be required for the surveillance behavior of CD8αα+ T-IELs, which includes both natural and induced T-IELs in mice, as observed through intravital microscopy [102]. It is not clearly understood whether this occurs through direct binding of a receptor on T-IELs to HVEM on IECs. However, for direct binding, potential ligands for the HVEM receptors include CD160 and LIGHT expressed on T-IELs [102]. Additionally, T-IELs also express GLP2R, the receptor for glucagon-like peptide 2 (GLP2), which regulates intestinal barrier function and suggests a role in epithelial maintenance [75,103,104]. GLP2 is produced by enteroendocrine L cells in the intestines [130]. GLP2R expression on T-IELs links these cells to intestinal lining maintenance and repair by enhancing barrier function, promoting epithelial proliferation, reducing mitochondrial damage, and decreasing apoptosis [103,104,105]. However, further research is needed to clarify these interactions.

In conclusion, microbial stimuli, in conjunction with proteins such as occludin, E-cadherin, and EpCAM on T-IELs, are crucial for establishing interactions with IECs. These interactions promote rapid T-IEL migration, regulate barrier permeability, and maintain epithelial integrity. HVEM ligands and GLP2R add layers of complexity by enhancing surveillance and maintenance functions. The interactions and roles of cell adhesion molecules have been extensively studied in TCRγδ+ T-IELs; however, whether TCRαβ+ T-IELs engage in similar interactions and the full spectrum of functions mediated by these interactions across different T-IEL subsets remain unclear. Future research could explore the mechanisms through which T-IELs, particularly TCRαβ+ subsets, contribute to surveillance and barrier maintenance.

### 2.5. Crosstalk Between T-IELs and the Enteric Nervous System

The potential crosstalk between T-IELs and the enteric nervous system (ENS) via the expression of neuropeptide receptors such as GPR171 and VIPR2 (vasoactive intestinal peptide receptor 2) as well as through neural cell adhesion molecules such as NCAM1 and NrCAM on T-IELs underscores a complex neuroimmune interface within the gut mucosa, integrating immune responses with neural activities. T-IELs express neuropeptide receptors such as GPR171 and VIPR2, indicating their responsiveness to neuroactive peptides released by the ENS [75]. GPR171 and VIPR2, which are known to bind to BigLEN and vasoactive intestinal peptide (VIP) respectively, can modulate immune responses [75]. BigLEN suppresses T cell activation and anti-tumor responses [131]. VIP is known to activate the cAMP/PKA pathway in conventional T cells for its anti-inflammatory properties [132,133,134]. Moreover, VIP is associated with Th2 cell development and inhibition of Th1 and Th17 responses [132,133,134]. The anti-inflammatory properties of VIP have been confirmed in knockout mouse models, where VIP-deficient mice develop lung inflammation [133,134]. Furthermore, TCRγδ+CD8αα+ T-IELs express the neural cell adhesion molecules NCAM1 (CD171) and NrCAM, which might play critical roles in homophilic adhesion and axonal guidance with the nerve endings [75]. Therefore, it is reasonable to speculate that these ligand-receptor interactions might be involved in communication between T-IELs and the enteric nervous system.

Future research could explore the interactions between T-IELs and the ENS, particularly focusing on how T-IEL-expressed neuropeptide receptors such as GPR171 and VIPR2 respond to neuroactive peptides and the importance of neural cell adhesion molecules such as NCAM1 (CD171) and NrCAM on T-IELs.

## 3. T-IEL Ligands in Diseases

During disease states, T-IELs engage with multiple ligand-receptor interactions in a sequential manner to mount effective immune responses (Figure 1; right). Initially, TCRγδ+ T-IELs respond through IEC’s PRR-dependent pathways, where IEC-derived IL-15 trigger T-IELs to produce antimicrobial peptides (AMPs) such as RegIIIγ [5,26,53,106,126,135,136]. This early response is followed by TCR-dependent mechanisms, particularly in TCRαβ+CD8αβ+ T-IELs, which recognize pathogen-derived peptides presented on MHC I molecules by IECs and respond with IFNγ production and cytotoxicity [137,138,139,140,141,142,143]. Moreover, co-receptor interactions provide additional layers of immune defense. For instance, NK receptors such as NKG2D and NKG2C on T-IELs interact with stress-induced ligands such as MICA/MICB and ULBP family members on infected cells to initiate cytotoxicity, a process that may occur independently of or in conjunction with TCR signaling [4,96,136,144,145,146]. The HVEM-CD160 interaction activates cytotoxic responses and promotes RegIIIγ production through the HVEM-Stat3-REG3 pathway [147,148]. OX40-OX40L signaling enhances cytokine production and cytotoxicity during infection [149]. In chronic inflammatory diseases, persistent IL-15 production by IECs leads to increased expression of NK receptors (NKG2D, NKG2C) on TCRαβ+CD8αβ+ T-IELs [96,98,150,151,152,153]. These T-IELs recognize stress ligands such as HLA-E and MICA on damaged epithelial cells, leading to cytotoxic activity that exacerbates epithelial damage and promotes further inflammation. Moreover, in cancers, TCRαβ+CD8αβ+ T-IELs can eliminate tumor cells expressing NKG2D ligands, such as MICA and ULBP, through FasL-mediated cytotoxicity [154], with the CD103/E-cadherin interaction facilitating contact-dependent killing [155], thereby contributing to the control of tumor progression and invasion. Furthermore, TCRγδ+ T-IELs utilize junctional adhesion molecule-like protein (JAML)-coxsackievirus and adenovirus receptor (CAR) and CD100-mediated pathways to promote epithelial healing through Keratinocyte Growth Factor (KGF) production [69,156].

This complex network of ligand-receptor interactions allows T-IELs to respond appropriately to various pathological conditions, maintaining intestinal homeostasis and promoting tissue repair when needed, as illustrated in Figure 1 (right).

### 3.1. Infectious Diseases

#### 3.1.1. Pathogen-Derived Ligands and T-IEL-Mediated Clearance

During infections, T-IELs coordinate IEC-dependent pathways [5,26,53,106,126,135,136], TCR-mediated responses [137,138,139,140,141,142,143,157], and co-receptor interactions [96,98,136,144,145,153] to effectively combat pathogens. The engagement of specific ligands with these corresponding receptors plays an important role in shaping intestinal immune defenses and facilitating pathogen clearance, ultimately influencing disease progression and outcomes.

##### Early Responses Mediated by Cytokine Receptor Signaling

TCRγδ+ T-IELs are major players in early intestinal immune responses, utilizing IEC-dependent, TCR-independent mechanisms through interactions with various epithelial microenvironment ligands to prevent pathogen colonization and preserve epithelial barrier functions. These T-IELs preferentially migrate to mucosal sites where pathogens adhere and generate a robust immune response [5,26,53,106,126,135,136].

The initial response of TCRγδ+ T-IELs to infections such as *Salmonella Typhimurium* and *Toxoplasma gondii* is independent of TCR signaling, which is demonstrated by the inability of TCR signaling inhibitors, targeting Zap70/Syk kinases, and TCR signaling-blocking antibodies to alter their migration and defensive responses during the infections. Specifically, TCRγδ+ T-IELs limit these infections through indirect signaling involving the PRRs on IECs, which initiates a MyD88-dependent pathway on IECs to stimulate the production of the RegIIIγ AMP from these T-IELs in IL-15-independent manner [5,26,53,106,126,135,136]. Moreover, *Salmonella* triggers IL-23 production by IECs in a TLR-dependent manner, which in turn stimulates TCRγδ+ T-IELs to produce IL-22 to limit the bacterial invasion by inducing the secretion of bactericidal Angiogenin 4 AMP by Paneth cells [158]. Apart from bacterial defense, IL-22 produced by TCRγδ+ T-IELs could also help to activate goblet cells to secrete mucus, which could prevent GI epithelia from larger pathogens including helminths [159]. This is evidenced by experiments where IL-22-deficient mice are unable to expel the *Nippostrongylus brasiliensis* and *Trichuris muris* from their intestines due to a reduced number of goblet cells and intestinal mucins [159,160].

Overall, TCRγδ+ T-IELs play an indispensable role in early intestinal immune responses, leveraging intestinal ligands-mediated and TCR-independent mechanisms to prevent pathogen colonization and maintain epithelial barrier functions. Although human peripheral TCRγδ+ T cells respond directly to microbial products through their TLR stimulation and also recognize bacterial phosphoantigens via their TCR [43,161,162,163], it is yet to be determined whether TCRγδ+ T-IELs are also capable of directly recognizing luminal microbes in such manners. Furthermore, the mechanisms through which TCRαβ+ T-IELs sense pathogens and initiate their early responses, particularly those mediated by cytokine receptor signaling, remain poorly understood. While IL-15 signaling is known to enhance the effector functions of antigen-primed TCRαβ+ T-IELs, which are all induced T-IELs, its role in primary infections has not been thoroughly investigated [164,165,166,167]. Understanding the cytokine receptor-mediated mechanisms by which T-IELs detect pathogens during early infections can be crucial for developing targeted therapies to enhance mucosal immunity and combat intestinal infections.

##### TCR-Dependent Mechanisms

TCR being the major antigen receptor on T cells, it is likely that TCR ligands are critical players in responding to and eliminating pathogens. Induced T-IELs including both TCRαβ+CD8αβ+ and TCRαβ+CD4+ subsets, are antigen-experienced and exhibit a strong response to TCR stimulation [38,75,168]. However, in case of new infections, antigen-specific T-IELs typically develop within at least seven days following the exposure [137,138,139,140,141,142,169]. Evidence predominantly supports the role of the TCRαβ+CD8αβ+ subset in recognizing intracellular pathogen-specific ligands and mediating immune responses [137,138,139,140,141,142,169]. TCRαβ+CD8αβ+ T-IELs are the ones known to detect various pathogen ligands presented on MHC I molecules by cells such as IECs, responding in an antigen-specific manner by producing cytokines such as IFNγ and resulting cytotoxicity of infected cells [137,138,139,140,141,142,143]. For instance, during *Encephalitozoon cuniculi* infection, the depletion of TCRαβ+CD4+ T-IELs does not alter the outcome, whereas the removal of TCRαβ+CD8αβ+ T-IELs leads to mortality of the infected animals [138], indicating a critical role of these T-IELs in pathogen clearance. Additionally, during *Encephalitozoon cuniculi* oral infection, TCRαβ+CD8αβ+ T-IELs demonstrate antigen-specific cytotoxicity as well as increased IFNγ production [138]. A similar response occurs during *Toxoplasma gondii* infections [139]. Notably, adoptive transfer of TCRαβ+CD8αβ+ T-IELs from infected mice provides long-lasting protection against *T.* gondii in an IFNγ-independent manner [139,140], indicating antigen-specific protective capacity. This specificity is also observed in responses to *Cryptosporidium* [141] and acute LCMV infections [142]. Moreover, although TCR signaling plays a critical role in the differentiation of TCRαβ+CD4+ T-IELs, their TCR-specific responses to pathogens remain unclear [79]. Once differentiated, these cells have been shown to react to antigens in the absence of cognate TCR interactions [79]. TCRγδ+ T-IELs have also been known to respond to virulent *Listeria monocytogenes* infections by producing IFNγ and exhibiting cytotoxicity in a TCR-dependent way. To further confirm, the depletion of their TCR using anti-γδ TCR monoclonal antibody GL3 diminished these responses [170]. However, the direct response of TCRγδ+ T-IELs to pathogens via TCR signaling remains less clear. Furthermore, peripheral TCRγδ+ T cells are known to directly recognize bacterial components such as Listeriolysin O and heat shock proteins through their TCR [31], suggesting a similar potential for TCRγδ+ T-IELs. 

Overall, TCR signaling plays a crucial role in T-IEL-mediated pathogen clearance, with TCRαβ+CD8αβ+ T-IELs being particularly effective in recognizing pathogen-specific ligands and mounting antigen-specific immune responses, while TCRγδ+ T-IELs also contribute to protection against certain infections through TCR-dependent mechanisms.

##### Co-Receptor Interactions

Ligands for T-IEL co-receptors such as NK receptors, CD160, and OX40, are essential for immune defense within the GI epithelia. NK receptor ligands are upregulated on T-IELs in response to infections, cellular stress, or malignant transformations, including MICA/MICB and ULBP family members in humans, and retinoic acid early inducible-1 (Rae1), histocompatibility 60 (H60), and UL16-binding protein-like Transcript 1 (MULT1) in mice [4]. TLR signaling in IECs upregulates these NK ligands and increases the IL-15 production, which in turn boosts the expression of co-stimulatory NK receptors such as NKG2D and NKG2C on T-IELs [96,144,145]. Additionally, the presence of inflammatory cytokines such as IFNγ and IL-1β in the GI microenvironment during infections further increases NKG2D expression on T-IELs [171,172]. Moreover, stress conditions upregulate the expression of NKG2C-associated DAP12 adapter protein in TCRαβ+CD8αβ+ T-IELs, enhancing their functional capabilities [96]. The interaction of NKG2D with its ligands significantly lowers the TCR activation threshold, enabling the recognition of non-cognate, low-affinity antigens or allowing operation independently of the TCR [98,145,153]. For instance, oral infection with *Salmonella enterica* serovar Typhimurium triggers the expansion of TCRγδ+CD8+ T-IELs. This response is accompanied by increased NKG2D expression on these T-IELs and upregulation of MULT1 on IECs, which enables T-IELs to destroy infected intestinal cells [136]. Blocking NKG2D or reducing these T-IELs decreases the clearance of pathogens such as *Salmonella* from the intestine and other tissues [136]. Furthermore, HLA-E molecules presenting pathogen-derived peptide ligands under stress conditions could further activate NKG2C, intensifying the cytotoxic responses of T-IELs during infections [146]. Apart from NK receptors, HVEM serves as a ligand for CD160 co-stimulatory receptor expressed on most T-IELs, the interaction that is essential for pathogen defense. This interaction induces a cytotoxic and inflammatory response in TCRαβ+CD8αβ+ T-IELs against pathogens in a TCR-dependent manner [147,173]. Moreover, this HVEM-CD160 interaction activates the HVEM-Stat3-REG3 pathway on IECs, which promotes the production of RegIIIy AMP by IECs to eliminate pathogens [148]. Similarly, intestinal LIGHT ligand, which is also expressed on T-IELs, binding to HVEM has been known to be crucial in defense against pathogens such as *Salmonella Typhimurium* [102], probably also functioning through the HVEM-Stat3-REG3 pathway on IECs. Additionally, TCR signaling increases the expression of OX40 co-stimulatory receptor on CD8+ T-IELs within 24 hours, otherwise absent under steady-state conditions, leading to pathogen clearance by enhancing cytokine production and cell-mediated cytotoxicity [149]. This study also noted the expression of both OX40 and its ligand OX40L on T-IELs [149], suggesting a mechanism to stimulate each other for pathogen clearance. Besides these co-receptors, it has been recently discovered that TCRαβ+CD4+CD8αα+ T-IELs can express TLR-4, a receptor not previously reported on T-IELs [45]. During sepsis, eCIRP from damaged cells binds to TLR-4, which triggers these T-IELs to produce granzyme B and perforins that lead to the death of IECs through cytotoxicity [45]. However, it is not yet known whether TLR-4 on TCRαβ+CD4+CD8αα+ T-IELs directly recognizes pathogen ligands such as LPS.

In summary, T-IELs play an important role in the immune defense of the intestinal mucosa by engaging IEC-dependent pathways, TCR-dependent mechanisms, and co-receptor interactions to combat various pathogens. Future research could focus on understanding the precise mechanisms by which T-IELs recognize pathogens, including the role of TCRs and the potential functions of PRRs on T-IELs in detecting specific PAMPs. Studies could also examine co-receptors activity in diseases, investigating changes in their expression during intestinal infections and inflammation, as well as the availability and distribution of their ligands in the intestinal epithelium. Investigating these mechanisms could lead to novel therapeutic strategies, including targeting TCR or co-receptor pathways and enhancing their ligand expression, to boost immune responses against intestinal pathogens and to improve clinical outcomes in GI diseases.

### 3.2. Chronic Diseases

T-IELs play a dual role in chronic illnesses, contributing to both disease progression and immune regulation depending on interactions between available ligands and expressed T-IEL receptors in the microenvironment. In chronic inflammatory conditions such as celiac disease (CeD) and inflammatory bowel disease (IBD), persistent IL-15 production by IECs induces major functional changes in T-IELs. IL-15 upregulates NK receptors, such as NKG2D, on TCRαβ+CD8αβ+ T-IELs [96,98,152,153]. These receptors recognize stress-induced ligands, including HLA-E and MICA, expressed on damaged IECs. This ligand-receptor interaction drives TCR-independent T-IEL activation, triggering a cytotoxic response that exacerbates inflammation and tissue damage [174,175,176,177,178,179]. Recruitment of T-IELs to inflammatory sites via the CXCR3/CXCL10 chemokine axis further amplifies inflammation and perpetuates disease progression [180]. In cancer, T-IELs exhibit both protective and pathogenic roles depending on the microenvironment. Stress-induced ligands such as MICA and ULBP on tumor cells are recognized by NKG2D on T-IELs, initiating FasL-mediated cytotoxicity and direct tumor killing [154]. Additionally, CD103 on T-IELs interacts with E-cadherin on tumor cells to retain T-IELs in the tumor microenvironment and enhance their anti-tumor ability [155]. However, subsets of T-IELs expressing co-inhibitory receptors such as PD-1 or inhibited through galectin-3-mediated LAG-3 signaling may promote tumor growth [181,182]. The balance between T-IEL-mediated tumor suppression and immune evasion plays a critical role in shaping cancer progression. Overall, T-IELs play a central role in chronic disease pathogenesis, with their functions shifting between maintaining immune homeostasis and driving pathology based on microenvironmental signals and ligand-receptor interactions.

#### 3.2.1. Celiac Disease

Celiac disease (CeD) is an immune-mediated enteropathy driven by an aberrant immune response to dietary gluten. The disease is strongly associated with HLA-DQ2 and HLA-DQ8, which present deamidated gluten peptides as foreign ligands to CD4+ T cells [183,184]. Once activated, these CD4+ T cells secrete pro-inflammatory cytokines such as IFNγ, leading to tissue destruction [150,151].

A hallmark of CeD is the chronic upregulation of IL-15 by IECs in the GI epithelia [150,151]. IL-15 promotes the activation of TCRαβ+CD8αβ+ T-IELs by enhancing the expression of NK receptors, such as NKG2C and NKG2D, and increasing transcription of the DAP10 adapter protein [96,98,152,153]. Activation of these NK receptors enables T-IELs to kill neighboring IECs expressing stress-induced ligands, such as HLA-E and MICA [11,152,153,185]. Elevated IL-15 levels also drive the proliferation of TCRαβ+CD8αβ+ T-IELs and increase their production of cytotoxic molecules, including granzyme A, granzyme B, and perforin [186]. These cytotoxic TCRαβ+CD8αβ+ T-IELs are the primary mediators of tissue damage in CeD, whereas TCRαβ+CD4+ T-IELs do not contribute to this effect [175,176,177,178,179]. Notably, the cytotoxic activity of TCRαβ+CD8αβ+ T-IELs in CeD does not rely on TCR recognition of gluten, as gluten-specific TCRαβ+CD8αβ+ T-IELs are not observed [174]. Some TCRαβ+CD8αβ+ T-IELs in CeD patients express the NKp46, a receptor that interacts with ecto-calreticulin (ecto-CRT), a stress ligand translocated to the cell surface under endoplasmic reticulum stress. IL-15 may further upregulate NKp46 expression on both TCRαβ+CD8αβ+ and TCRγδ+ T-IELs, amplifying their cytotoxic responses [4]. Additionally, IECs secrete CXCL10, a chemoattractant that recruits CXCR3-expressing TCRαβ+CD8αβ+ T-IELs. This CXCR3/CXCL10 axis is hyperactivated in the small intestinal mucosa of untreated CeD patients, exacerbating tissue damage [180]. Moreover, CeD involves an increase in inflammatory TCRγδ+ T-IEL population [187,188]. Gluten exposure causes a loss of IEC-expressed BTNL3/8 and the resident Vγ4+ T-IELs, which are replaced by pro-inflammatory IFNγ-producing Vγ3+ T-IELs [189,190]. This shift is not reversed by a gluten-free diet, indicating that chronic inflammation may permanently reconfigure the tissue-resident TCRγδ+ T-IEL compartment, disrupting protective IEC-T-IEL interactions [190]. In addition, TCRγδ+CD8+ T-IELs that suppress the cytotoxic programming of TCRαβ+ T-IELs via CD94/NKG2A engagement and TGFβ production, are reduced in active CeD [191].

Overall, CeD is characterized by complex immune responses driven by interactions between T-IELs and IECs. Key ligands contributing to intestinal damage and disease pathology include dietary gluten, cytokines such as IL-15, and stress-induced molecules like MICA. These factors collectively drive epithelial destruction, with TCRαβ+CD8αβ+ T-IELs playing a central role through their recognition and response to these ligands.

#### 3.2.2. Inflammatory Bowel Disease

Inflammatory bowel disease (IBD) refers to Crohn’s disease and ulcerative colitis, two chronic inflammatory diseases of the GI tract that may involve inappropriate immune responses against intestinal microbes [43]. As the lesions in IBD can be transmural, affecting the entire thickness of the bowel wall that completely removes the epithelial layer where T-IELs reside [173], it might be that the primary role of T-IELs is more in the prevention of this disease and may also contribute to its resolution, particularly through immunoregulation and healing functions. IBD shares many similarities with CeD such as overexpression of IL-15 from IECs [69]. Also, disease severity correlates with an increased number of TCRγδ+ cells in the intestinal mucosa [192,193]. Moreover, decreased levels of Galectin-3 are associated with IBD [194,195,196], which may reduce LAG-3 co-inhibitory signaling from T-IELs, aiding inflammation. In addition, a low amount of Vitamin A, Vitamin D, and AhR has been associated with IBD [43,123]. These ligands are involved in the gut migration and maintenance of T-IELs, which indicates that the low numbers of T-IELs might lead to IBD. Despite these observations, the role of T-IELs in IBD remains complex and not fully elucidated. Recent studies have reported conflicting results regarding the presence of CD4+CD103+ T cells in IBD patients [197,198], while a decrease in CD8αβ+CD103+ T cells has been observed in inflamed mucosa of these patients [198]. CD4+CD103+ cells in IBD patients exhibit increased production of pro-inflammatory cytokines, such as IFNγ, IL-17A, and TNFα [199,200]. In contrast, CD8+CD103+ cells, although reduced in number, express regulatory molecules such as IL-22, IL-26, and CCL20 [201], suggesting distinct functional roles. However, it is important to note that both T-IELs and lamina propria T cells can express CD103, making it challenging to distinguish between these populations. For instance, CD103 is expressed on > 90% of T-IELs and 45–50% of lamina propria T lymphocytes [202].

Future studies could investigate how T-IEL populations change during IBD compared to healthy conditions and how these shifts influence inflammation and tissue repair. Understanding the availability of key ligands and their roles in T-IEL development and function within inflamed tissue is essential. Additionally, examining the receptors expressed by T-IELs and their interactions with their ligands could provide insights into their functions. These findings could help develop targeted therapies to restore immune balance and promote tissue repair in IBD.

#### 3.2.3. Cancer

The major ligands known to be responsible for triggering immune responses in intestinal cancers such as colon cancer are stress-induced ligands expressed on these cells, such as MICA and ULBP, which is similar to that in other chronic diseases such as CeD and IBD. This is why NK receptors play a crucial role in targeting and killing these malignant cells. For example, human TCRαβ+CD8αβ+ T-IELs can spontaneously kill colon cancer cell lines that express NKG2D ligands by inducing FasL-mediated cytotoxicity and producing inflammatory cytokines such as TNFα and IFNγ [154]. Additionally, natural TCRαβ+CD8αα+ and TCRγδ+ T-IELs can kill tumor cells independently of TCR activation, through the stimulation of NKR-P1 cell receptors [203]. Moreover, TCRγδ+ T-IELs that express the NKp46 receptor demonstrate strong cytotoxicity against colorectal cancer (CRC) and help reduce the risk of metastasis [204]. The use of co-receptors by T-IELs to eliminate the tumor is further supported by the fact that in a small intestinal tumor organoid model, the T-IELs eliminated these intestinal tumors in cell-to-cell contact dependent-manner through CD103/E-cadherin signal [155]. Additionally, Galectin-3, which is overexpressed in various types of epithelial cancers including CRC, is associated with decreased epithelium integrity in human colon cancer cells [182]. This may be partly due to co-inhibitory signaling through LAG-3 in T-IELs. In contrast, PD-1- and IL-17-expressing Vγ4+ and Vγ6+ T-IELs exhibit pro-tumorigenic functions, indicating the role of the PD-1 co-receptor and IL-17 in supporting cancer progression [181]. 

Overall, besides the stress ligands that engage NK receptors, the specific ligands of T-IELs that influence anti-tumor or pro-tumor conditions are largely unknown. Additionally, there is limited evidence of TCRαβ+CD4+ T-IELs’ role in intestinal epithelial tumor microenvironment. Future research could focus on identifying the expression of different cancer-related receptors on T-IEL subsets and the ligands in the cancer microenvironment that can modulate the anti-tumorigenic and pro-tumorigenic activities of specific T-IEL subsets. Clarifying these mechanisms could lead to targeted therapies that help manipulate these interactions to combat intestinal cancers more effectively.

### 3.3. Epithelial Repair and Healing

TCRγδ+ T-IELs utilize the junctional adhesion molecule-like protein (JAML) and CD100 pathways to stimulate key interactions crucial for epithelial healing. These ligand-receptor interactions facilitate Keratinocyte Growth Factor (KGF) production and maintenance of the epithelial barrier.

TCRγδ+ T-IELs express the co-stimulatory molecule, JAML, which binds to coxsackievirus and adenovirus receptor (CAR) on IECs, resulting in the upregulation of KGF, a critical factor for IEC proliferation and epithelial healing by facilitating the repair and maintenance of the intestinal epithelium [69]. CAR is part of the epithelial tight junction and aids interactions between IECs [205]. The condition under which CAR on the IECs binds to JAML on TCRγδ+ T-IELs to trigger epithelial repair and healing via KGF production remains unclear. Moreover, the expression of CD100, a co-receptor expressed on all colonic T-IELs, has been found to be important for KGF production, as TCRγδ+ T-IEL deficient in CD100 cannot produce this molecule [156]. However, KGF production could not be stimulated by CD100 cross-linking on TCRγδ+ T-IEL in vitro, suggesting that CD100 influences TCRγδ+ T-IEL functions in conjunction with other signals [156].

Overall, the restoration and maintenance of the intestinal epithelium are facilitated by critical ligand-receptor interactions. The JAML-CAR interaction notably stimulates KGF production, which is a key factor in epithelial healing [69]. Despite the importance of the CD100 co-receptor in this process, its full impact remains dependent on additional signaling pathways, which are yet unknown. Moreover, the healing and repair role of other T-IELs besides TCRγδ+ T-IELs has not been reported yet. Future studies could focus on understanding these complex ligand interactions in TCRγδ+ T-IELs and also understanding the role of TCRαβ+ T-IELs on healing and maintenance of the epithelial barrier to develop targeted therapies that enhance epithelial repair in diseases such as chronic intestinal inflammatory conditions.

## 4. Conclusions

In conclusion, this review highlights the complex interplay between T-IEL receptors and their corresponding ligands in the intestinal epithelium. The intricate network of ligand-receptor interactions, including TCRs and non-TCRs, such as co-inhibitory and co-stimulatory receptors, cytokine receptors, nuclear receptors, and various signaling molecules, directs T-IEL functions in both steady-state and pathological conditions. The availability of ligands, coupled with the differential expression of receptors on T-IELs within the GI microenvironment, enables these cells to maintain immune tolerance, effectively monitor and eliminate pathogens, infected or damaged cells, and malignancies, as well as contribute to tissue repair and healing.

## Data Availability

No new data was created for this review.
